# QuickStats

**Published:** 2014-09-05

**Authors:** FROM THE NATIONAL CENTER FOR HEALTH STATISTICS

**Figure:**
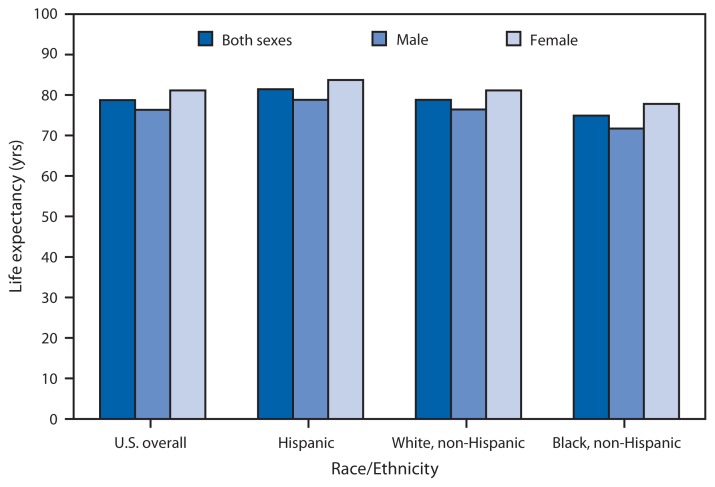
Life Expectancy at Birth, by Sex and Race/Ethnicity — United States, 2011

In 2011, life expectancy at birth was 78.7 years for the total U.S. population, 76.3 years for males, and 81.1 years for females. Life expectancy was highest for Hispanics for both males and females. In each racial/ethnic group, females had higher life expectancies than males. Life expectancy ranged from 71.7 years for non-Hispanic black males to 83.7 years for Hispanic females.

**Source:** National Center for Health Statistics. Deaths: final data for 2011. Available at http://www.cdc.gov/nchs/data/nvsr/nvsr63/nvsr63_03.pdf.

**Reported by:** Arialdi Minino, aminino@cdc.gov, 301-458-4376.

